# Radiation oncology department policy development for patients who may become pregnant

**DOI:** 10.1002/acm2.14256

**Published:** 2024-01-04

**Authors:** Jessica M. Fagerstrom

**Affiliations:** ^1^ Department of Radiation Oncology University of Washington Seattle Washington USA; ^2^ Department of Radiation Oncology Kaiser Permanente Seattle Washington USA

**Keywords:** consent, embryo, fetal radiation, fetus, policy, pregnancy, radiation therapy

## Abstract

In the context of radiation oncology, radiation exposure from radiation therapy simulation, image guidance, and radiation therapy procedures can have severe adverse biological effects on a developing embryo or fetus. Patients who may be pregnant are screened for the possibility of pregnancy to prevent unnecessary or excessive exposure of radiation in utero. Some radiation therapy patients for whom a pregnancy test is indicated may elect to decline the test. In addition, some patients who are found upon screening to be pregnant may decide, with their attending radiation oncologist, to continue with treatment. A radiation oncology department policy was developed to provide guidelines regarding screening and consent. The policy was designed to prevent unnecessary exposure to patients who may be pregnant, and to limit dose to the embryo or fetus in patients for whom treatment is medically indicated. The policy is presented as an example for physicists intending to develop or revise their own practice's policy regarding patients who may become pregnant.

## 1 INTRODUCTION

Because radiation therapy delivered to pregnant patients poses a risk to the developing embryo or fetus, it is important for radiation oncology departments to screen patients, prior to initiating care, who may become pregnant. A national survey of Canadian radiation therapists, medical physicists, and radiation oncologists found that approximately one fifth of respondents had experienced an event in which a pregnant patient received or almost received radiation therapy without protection measures in place.^1^ According to AAPM's MPPG 11.A,^2^ pregnancy status is one of the key items of patient information recommended to be verified by simulation therapists, and then reviewed as a component of physics initial plan quality assurance. TG‐275 indicates that failure modes regarding patient assessments, including miscommunications regarding patient pregnancy status, represent a high‐risk failure mode for initial plan and chart quality assurance.^3^ However, many radiation oncology departments do not systematically or routinely verify patient pregnancy status. A 2021 study noted that ASTRO radiation oncologists responding to an electronic survey indicated that they would “strongly agree” or “agree” that screening for pregnancy should be done prior to delivery of radiation therapy (84.8% of respondents), but 29.7% of respondents noted that their department did not have such a screening policy in place, and 7.1% of respondents indicated that their department did not screen for pregnancy.^4^ The same survey indicated that having a departmental policy in place for screening for pregnancy was strongly correlated with respondents screening for pregnancy (*p* = 0.0005). These results echo those of a 2016 survey of National Comprehensive Cancer Network member institutions, which found that 30% of respondents did not have a guideline or policy in place on screening patients for pregnancy prior to treatment via radiation therapy, surgery, or chemotherapy.^5^

As noted by the NRCP, irradiation of pregnant patients can have severe effects on the developing embryo or fetus, including lethality, cancer, microcephaly, decreased intelligent quotient, epilepsy, congenital malformations, and neurobehavioral effects.^6^ These effects are dependent on multiple factors, including the stage of the developing embryo or fetus at the time of irradiation, and the absorbed dose. The ICRP states that although stochastic effects do not have an identifiable threshold dose, effects due to cell killing maintain a threshold of approximately 10−20 cGy.^7^ The ICRP values correspond well with the recommendation in AAPM's Task Group 36 report,^8^ to attempt to keep fetal dose to < 10 cGy to minimize risk. It is important to note the limitations in accuracy from the dose estimates included in TG‐36, as shown by Kry et al.^9^ Through computational and empirical methods, previous work has evaluated peripheral dose to the fetus when treating pregnant patients, both with and without custom shielding in place (see, e.g., Prado et al.,^10^ Bednarz and Xu,^11^ Owrangi et al.,^12^ and Labby et al.^13^).

Note that some patients for whom a pregnancy test is indicated may elect to decline the test. While there exists a need for thorough pregnancy screening in the radiation oncology setting, departments also have the requirement to protect patient autonomy in medical decision making. Further, after consulting with their attending physician, patients who are screened for pregnancy who are found to be pregnant may elect to terminate the pregnancy, postpone treatment until after delivery or until a later gestational age is reached, or continue with radiation therapy during pregnancy. As stated in the AAPM's Code of Ethics, AAPM members have a responsibility to “respect the autonomy and dignity of all patients”.^14^ Patients must maintain the right to participate in the informed consent process, to refuse services, and to balance medical necessity with risk.^15^

Based on this information, a radiation therapy department in a US community hospital setting sought to formalize a more extensive policy as a quality improvement initiative, regarding patients who may become pregnant. This department had already screened patients for possible pregnancy, but desired a more comprehensive policy, including arrangements for patients who decline to take a pregnancy test. This department offers external beam and brachytherapy services, and routinely treats patients who meet screening criteria for patients who may become pregnant. The revised, resulting policy is described in the following section.

## 2 METHODS

**FIGURE 1:**
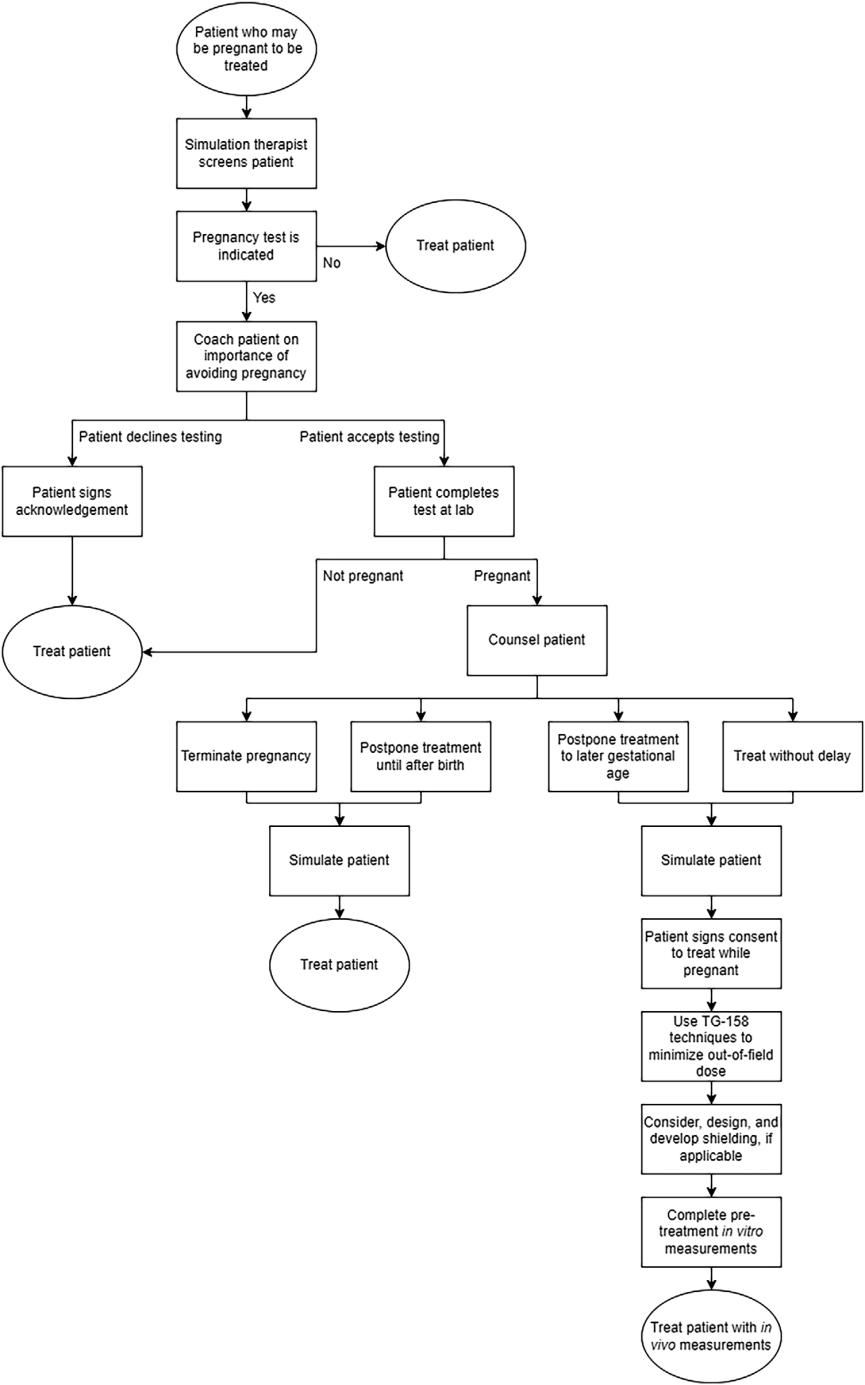
Flowchart of departmental policy for managing the care of patients who may become pregnant. Only pathways that result in patient treatment are included.

Details of the department's revised and formalized workflow are as follows (and illustrated in Figure 1): all patients ages 12−50 with an intact uterus are asked by the simulation therapist about the possibility of pregnancy. Radiation therapy and radiation therapy CT simulation, even if not involving the abdomen or pelvis, still pose radiation exposure risk, and these patients should still be screened for possible pregnancy. If the patient has had a hysterectomy or bilateral oophorectomy, or meets the definition for medical postmenopause (the patient has not experienced menstruation for at least 12 consecutive months associated with no other medical cause such as hormonal birth control^16^), then this will be documented in the patient's chart, and a pregnancy test is not indicated.

Prior to CT simulation, patients for whom a pregnancy test is indicated will be asked to visit the hospital laboratory for a pregnancy test. If the patient complies with pregnancy testing, results of the test will be imported into the department's record and verify system. The CT simulation process will not be completed until the results are obtained by the department. If a patient for whom a pregnancy test is indicated declines the test, they will be counseled on the risks associated with continuing the treatment simulation and treatment procedures. They will sign an acknowledgement with the following language (adapted with minor changes from Appendix B of AAPM's TG‐36^8^):

I confirm that I am not pregnant and that I will not become pregnant over the course of my treatment. Not all patients are able to become pregnant. I understand that it is recommended that all patients who may be pregnant take a pregnancy test prior to radiation therapy simulation, treatment planning, and radiation therapy treatment delivery. I have been offered the opportunity to take a pregnancy test, and I decline. If I am pregnant or become pregnant during my care, I understand that radiation can be harmful to an unborn child. There is a possibility of miscarriage and there is also the possibility that the child may not develop or grow in a normal manner because of radiation exposure. I have had an opportunity to discuss these matters with my physician and to ask questions about my condition, alternative methods of treatment, and the proposed procedure(s).

This acknowledgement is in addition to the standard consent process completed during initiation of care for all patients in the department.

If the patient is pregnant at the time of the initial pregnancy test, or if the patient becomes pregnant over the course of their simulation and treatment, the patient may decide to decline radiation therapy, terminate the pregnancy and receive radiation therapy, postpone treatment until after delivery or until a later gestational age is reached, or continue with radiation therapy during pregnancy. Note that Figure 1 only includes pathways in which the patient receives radiation therapy. If the attending physician and the patient elect to continue treatment during pregnancy, the course of treatment will be impacted by the pregnancy. In this case, after the patient receives appropriate counseling from the physician, they will sign a consent to continue treatment while pregnant. Modifications of treatment techniques may be used to lessen in utero exposure of the embryo or fetus to radiation by minimizing out‐of‐field dose,^17^ including the following techniques:

- reducing field size and treatment volumes,
- adjusting beam energy,
- using flattening‐filter‐free beams,
- orienting the MLC leaves open along the axis of the patient instead of across the patient,
- minimizing MU,
- using low modulation,
- excluding wedged fields,
- eliminating any field that approaches the vertex position, and
- using brachytherapy instead of external beam radiation.

The attending radiation oncologist will add documentation to the patient's treatment summary in the hospital's electronic health records system that states the rationale for treating while pregnant, and any relevant radiation exposure mitigation strategies. Following simulation and treatment planning, pretreatment in vitro measurements using an ion chamber and thermoluminescent dosimeters (TLDs) will be completed on an anthropomorphic phantom to estimate doses at four positions, as described in TG‐36^8^ and Labby et al.^13^ Measurements at the uterine fundus at its position at the time of simulation, the uterine fundus at its projected position at the conclusion of treatment, the umbilicus, and the pubic symphysis will help determine the expected estimated range of doses to the embryo or fetus. Specialized shielding may be designed. If a custom shield (such as those described by Owrangi et al.^12^ and Kang et al.^18^) is built or obtained temporarily from another facility, the patient will be counseled on any risks involved in using the equipment, as mobile shielding and bridge‐over‐patient designs may involve a large mass of lead. If shielding is to be used, pretreatment in vitro measurements will be completed prior to treatment both with and without shielding in place. Physicists will include special physics consult documentation in the department's record and verify system detailing how recommendations from AAPM's TG‐36 were followed,^8^ including modifications of the treatment plan, in vitro pretreatment measurements, the consideration of shielding, etc. The special physics consult document will then be approved by the physics team and the attending radiation oncologist. In vivo measurements will be required to estimate dose to the embryo or fetus on the first fraction. The physics team and the attending radiation oncologist may determine that additional in vivo dose assessments may be required throughout the treatment course, which will be documented in the patient's chart.

If a patient is found to be pregnant during the course of treatment after irradiation without protective measures, the incident will be documented within the institution's unusual occurrence and incident learning system, and the Radiation Safety Officer (RSO) and lead medical physicist will be notified. All available information will be given to the physicist to estimate the fetal dose. In vitro phantom measurements, as described previously, may be required. The physicist will provide recommendations that may be used for the RSO and attending physician to counsel the patient. The RSO will generate a memo to be included in the patient's chart. The following information will be included in the incident report:

- patient's name,
- patient's medical record number,
- approximate number of weeks patient is pregnant,
- date of exposure(s),
- estimated fetal dose,
- other pertinent information, if known; for example: exposure energy, MU, number of fractions, imaging studies, etc.

The RSO will comply with federal and state reporting requirements.

## 3 RESULTS

The physics group drafted a policy, which was reviewed by the multidisciplinary Radiation Oncology Quality Assurance Committee (ROQAC). After some minor changes, the policy was adopted by the department and sent to the hospital for inclusion in the institution's policy and procedure library. Language for the form used for patients to document declining a pregnancy test was drafted directly from AAPM's TG‐36,^8^ as well as comments from the committee. This language was approved by the department and then forwarded to the institution's Health Information Management Department for review and approval. As part of this approval process, the Health Information Management Department then coordinates review with the institution's Ethics and Compliance Program, which functions to ensure an ethical work environment in compliance with legal and regulatory requirements. At the time of this writing, two patients who met screening criteria declined pregnancy testing. No pregnant patients have been treated. Following the policy changes, the ROQAC revisited the project at the next available meeting. Committee members recommended continuing with the revised policy.

## 4 DISCUSSION

The policy takes into account that sometimes it is desirable to treat pregnant patients using radiation therapy, while minimizing peripheral dose. In these situations, medical necessity must be balanced with risk. Because risk to the developing embryo or fetus from ionizing radiation is in part dependent on the stage of the embryo or fetus at the time of irradiation, it may be possible to delay treatment to allow for the pregnancy to reach a more advanced gestational age when risk to the pregnancy is lower.

As discussed in the policy, in the event that a pregnant patient is treated accidentally without protection measurements in place (e.g., it was not known at the time of treatment that the patient was pregnant, but is discovered later), it is necessary to comply with state and federal regulations. For example, the NRC requires that licensees report doses >50 mSv to an embryo or fetus from byproduct material unless the dose was approved in advance by the authorized user.^19^ The hospital for which this policy was developed would rely on the radiation safety office for all reporting requirements; however, physicists developing or revising their own department policies should consult with applicable regulations if they will be responsible for all reporting.

The approach as described in this technical note is based on the specific workflows of the institution adopting the policy. Note that as discussed in AAPM's TG‐100 report,^20^ a policy should be designed based on the particular work environment for which it is intended to be used. Physicists interested in adopting a similar policy at their own institutions should evaluate required changes based on their specific work environment. They are also encouraged to discuss further considerations not explicitly addressed by the policy with their own risk assessment team. A full TG‐100 analysis was not completed when revising this example policy, because a complete formal process tree was not determined with the entire multidisciplinary team. However, the ROQAC did discuss severity, incidence rates, and lack of detectability of a selection of failure modes, such as if a patient were to become pregnant during the course of treatment (following an initial negative pregnancy test). Although patients are counseled on the need to avoid pregnancy during treatment, this policy, as written, does not require ongoing serial pregnancy screening throughout a patient's treatment course. It would be possible for a patient to test negative for pregnancy at the time of initial screening, but then to conceive while on treatment. This concern was discussed at the department's multi‐disciplinary quality improvement meeting, and deemed to have a low expected rate of occurrence with a low overall risk priority number.

In the United States, different states maintain varying legal restrictions regarding reproductive health care. It is noted that this policy was written for use in an institution located within a state with legal protections in place for healthcare workers who provide legal health care services. This state protects access to sexual and reproductive health care, protects qualified healthcare workers providing care, and protects a pregnant individual's right to abortion services prior to viability or to protect the pregnant individual's life or health. Other states have different legal restrictions regarding reproductive health care. Further, there is legislation involving reproductive health that is currently pending litigation, and it is expected that legislation may continue to evolve. For these reasons, it is strongly encouraged to involve the institution's legal team when drafting documentation and policies.

The rate of cancer incidence is estimated at approximately 1 in 1000 pregnant patients, and approximately 4000 pregnant patients undergo radiation therapy per year in the United States.^21^, ^22^, ^23^ Though treating pregnant patients is not routine, it is advised for radiation oncology departments to implement policies regarding screening and treating patients who may become pregnant. For this work, a more detailed departmental policy was developed, reviewed, and instituted to replace a less formalized practice. Interested physicists are encouraged to review their own institution's policy for pregnant patients and consider adapting the policy presented here as appropriate.

## CONFLICT OF INTEREST STATEMENT

There are no conflicts of interest to disclose.

## Data Availability

All data generated or analyzed during this study are included in this published article.
